# Radiology AI Deployment and Assessment Rubric (RADAR) to bring value-based AI into radiological practice

**DOI:** 10.1186/s13244-023-01599-z

**Published:** 2024-02-05

**Authors:** Bart-Jan Boverhof, W. Ken Redekop, Daniel Bos, Martijn P. A. Starmans, Judy Birch, Andrea Rockall, Jacob J. Visser

**Affiliations:** 1 Erasmus School of Health Policy and Management, Erasmus University Rotterdam, Rotterdam, The Netherlands; 2 Department of Epidemiology, Erasmus University Medical Centre, Rotterdam, The Netherlands; 3 Department of Radiology & Nuclear Medicine, Erasmus University Medical Centre, Rotterdam, The Netherlands; 4 Pelvic Pain Support Network, Dorset, UK; 5 Department of Surgery & Cancer, Imperial College London, London, UK

**Keywords:** Value framework, Artificial intelligence, Evidence-based medicine, Local assessment, Value-based radiology

## Abstract

**Objective:**

To provide a comprehensive framework for value assessment of artificial intelligence (AI) in radiology.

**Methods:**

This paper presents the RADAR framework, which has been adapted from Fryback and Thornbury’s imaging efficacy framework to facilitate the valuation of radiology AI from conception to local implementation. Local efficacy has been newly introduced to underscore the importance of appraising an AI technology within its local environment. Furthermore, the RADAR framework is illustrated through a myriad of study designs that help assess value.

**Results:**

RADAR presents a seven-level hierarchy, providing radiologists, researchers, and policymakers with a structured approach to the comprehensive assessment of value in radiology AI. RADAR is designed to be dynamic and meet the different valuation needs throughout the AI’s lifecycle. Initial phases like technical and diagnostic efficacy (RADAR-1 and RADAR-2) are assessed pre-clinical deployment via in silico clinical trials and cross-sectional studies. Subsequent stages, spanning from diagnostic thinking to patient outcome efficacy (RADAR-3 to RADAR-5), require clinical integration and are explored via randomized controlled trials and cohort studies. Cost-effectiveness efficacy (RADAR-6) takes a societal perspective on financial feasibility, addressed via health-economic evaluations. The final level, RADAR-7, determines how prior valuations translate locally, evaluated through budget impact analysis, multi-criteria decision analyses, and prospective monitoring.

**Conclusion:**

The RADAR framework offers a comprehensive framework for valuing radiology AI. Its layered, hierarchical structure, combined with a focus on local relevance, aligns RADAR seamlessly with the principles of value-based radiology.

**Critical relevance statement:**

The RADAR framework advances artificial intelligence in radiology by delineating a much-needed framework for comprehensive valuation.

**Keypoints:**

• Radiology artificial intelligence lacks a comprehensive approach to value assessment.

• The RADAR framework provides a dynamic, hierarchical method for thorough valuation of radiology AI.

• RADAR advances clinical radiology by bridging the artificial intelligence implementation gap.

## Introduction

A survey among European Society of Radiology (ESR) members indicated a promising role for artificial intelligence (AI) in radiology, with over 50% of respondents using or considering its use [1]. Promises surrounding AI have been monumental, with the alleged enhancement of technical performance, detection, and quantification of pathologies to streamline radiologists’ workflows and improve patient outcomes [2–5]. AI has promised to generate value in image acquisition, preprocessing, and interpretation in various imaging modalities (CT, MRI, X-ray, and ultrasonography), but also in administrative radiology tasks that leverage generative AI.

This alleged value of radiology AI should, however, first be rigorously assessed before implementation. The general lack of knowledge regarding radiology AI’s added value has been reported elsewhere [2, 6, 7] and calls for a robust valuation framework to properly assess the value. The valuation must move beyond conventional metrics like sensitivity and specificity, delving into actual added value on a clinical level by considering among others patient impact, influence on clinical decision-making, workflow implications [8–12], and actual value for the patient.

In this paper, we present the Radiology AI Deployment and Assessment Rubric (RADAR), a framework designed to conceptualize the value of radiology AI across its entire lifecycle. Rooted within the widely endorsed concept of value-based radiology, RADAR emphasizes the centrality of patient outcomes [8, 13, 14]. Subsequently, we discuss various study designs that help to assess value in alignment with the distinct levels of RADAR.

## Radiology AI Deployment and Assessment Rubric (RADAR)

**Fig. 1:**
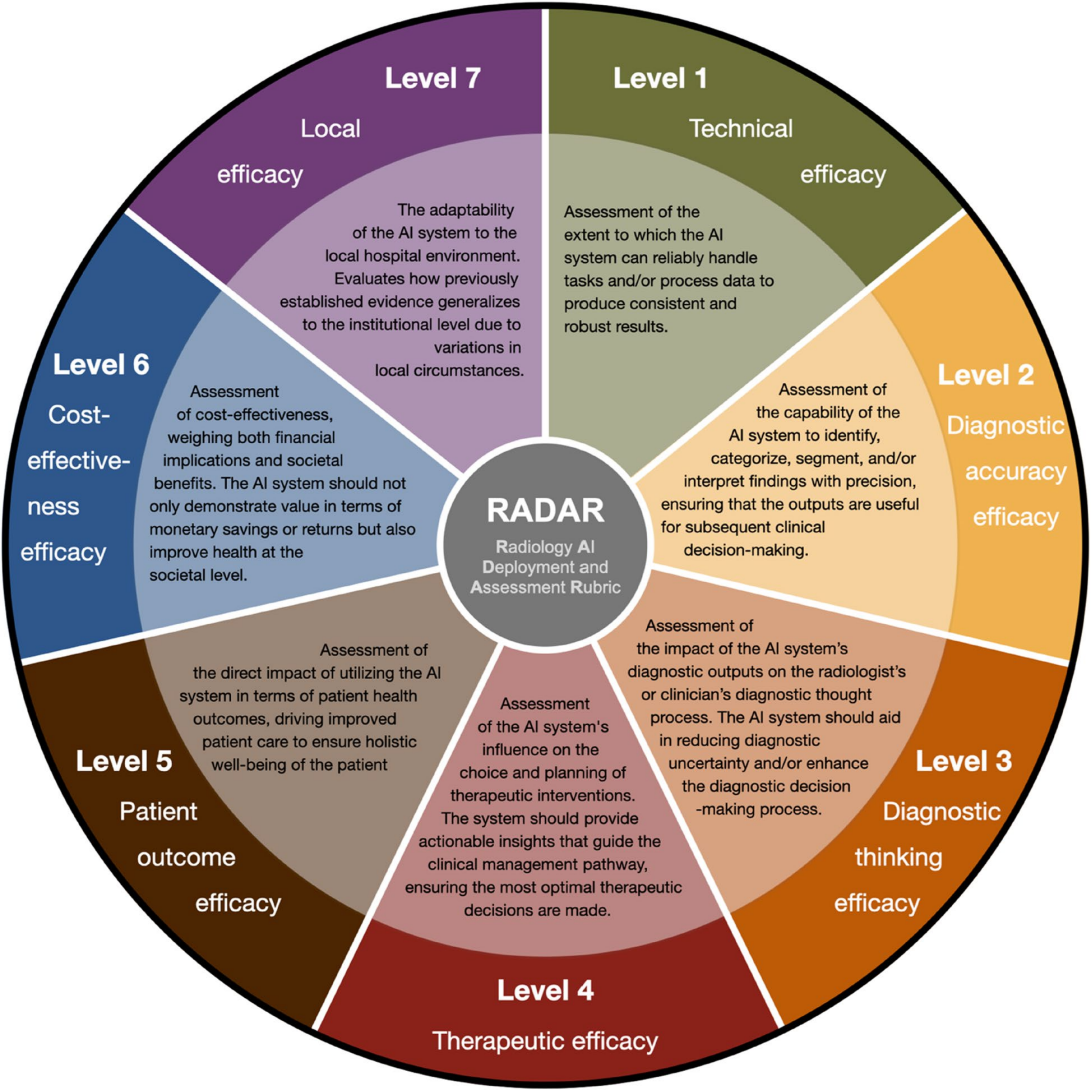
Overview of the RADAR framework. The outer circle depicts the RADAR efficacy level, and the inner circle provides its description. *Abbreviations*: AI, artificial intelligence; RADAR, Radiology AI Deployment and Assessment Rubric

The conceptual RADAR framework is depicted in Fig. 1. Table 1 provides a comprehensive definition of the various RADAR levels and links it to the various study designs discussed throughout this paper. RADAR is an adaptation of Fryback and Thornbury’s 1991 “Imaging Efficacy Framework” [10], designed to evaluate the efficacy of imaging technologies. It methodically progresses through seven hierarchical levels of efficacy, from specific to broader. Each efficacy level is foundational for the next: e.g., when technical efficacy (RADAR-1) is not ensured, progression to subsequent levels becomes redundant. We introduce the novel level of “local efficacy” (RADAR-7), underscoring the need for the valuation of an AI system within the local context. This is crucial, as insights from RADAR-1–6 might not translate universally across different healthcare institutions.

**Table 1:** RADAR definition, illustration, and connection to the relevant study designs

RADAR level	Definition	Illustration	Relevant study design
1. Technical efficacy	Assessment of the extent to which the AI system can reliably handle tasks and/or process data to produce consistent and robust results	The ability of an AI system to successfully process, analyze, and perform the relevant task on radiological images	*• Cross-sectional study* to evaluate AI processing speed and image handling *• *In silico* clinical trial* to evaluate AI processing speed and image handling in a simulated setting
2. Diagnostic accuracy efficacy	Assessment of the capability of the AI system to identify, categorize, segment, and/or interpret findings with precision, ensuring that the outputs are useful for subsequent clinical decision-making	The ability of an AI system to correctly diagnose pathologies (e.g., in terms of sensitivity and specificity)	*• Cross-sectional study* to evaluate AI accuracy on a validation set of images *• *In silico* clinical trial* to evaluate AI accuracy on a validation set of images in a simulated setting
3. Diagnostic thinking efficacy	Assessment of the impact of the AI system’s diagnostic outputs on the radiologist’s or clinician’s diagnostic thought process. The AI system should aid in reducing diagnostic uncertainty and/or enhance the diagnostic decision-making process	The capability of an AI system to optimize the radiologist’s diagnostic process (e.g., by taking away uncertainty in difficult diagnoses)	*• *In silico* clinical trial* to evaluate the AI system’s influences radiologists’ behavior in a simulated setting *• Randomized controlled trial* to evaluate the AI system’s influences radiologists’ behavior in a controlled setting
4. Therapeutic efficacy	Assessment of the AI system’s influence on the choice and planning of therapeutic interventions. The system should provide actionable insights that guide the clinical management pathway, ensuring the most optimal therapeutic decisions are made	The capacity of an AI system to influence and augment therapeutic decisions (e.g., by resulting in an increase in the number of surgeries performed)	*• Randomized controlled trial* to evaluate the quality of AI-driven therapeutic decisions in a controlled setting
5. Patient outcome efficacy	Assessment of the direct impact of utilizing the AI system in terms of patient health outcomes, driving improved patient care to ensure holistic well-being for the patient	The ability of an AI system to influence patient outcomes (e.g., in terms of life-years gained, length of stay, patient well-being)	*• Randomized controlled trial* to evaluate the AI system’s impact by comparing patient outcomes across treatment and exposure arms (e.g., in terms of life-years or QALYs) *• Cohort study* to evaluate the AI system’s impact by comparing exposed and non-exposed groups on patient outcomes
6. Cost-effectiveness efficacy	Assessment of cost-effectiveness, weighing both financial implications and societal benefits. The AI system should not only demonstrate value in terms of monetary savings or returns but also improve health at the societal level	The capacity of an AI system to optimize outcomes while minimizing societal costs, evaluated by contrasting long-term benefits (e.g., QALY’s gained) and incremental costs of AI adoption	*• Health economic evaluation,* such as cost-utility analysis (CUA), to evaluate AI system’s cost-effectiveness
7. Local efficacy	The adaptability of the AI system to the local hospital environment. Evaluates how previously established evidence generalizes to the institutional level due to variations in local circumstances	The extent to which an AI system’s efficacy generalizes to the unique hospital settings (e.g., in terms of workflow, infrastructure, patient demographics)	*• Budget impact analysis* to assess the impact on the local budget of adopting the AI system *• Multi-criteria decision analysis* to enable local decision-makers to consider diverse criteria for informed AI adoption *• Prospective monitoring* to ensure long-term local efficacy

*Abbreviations*: *AI* Artificial intelligence, *CUA* Cost-utility analysis, *QALY* Quality-adjusted life year, *RADAR* Radiology AI Deployment and Assessment Rubric

We illustrate RADAR with the hypothetical case of a multifunctional AI system for stroke care. RADAR commences with *technical efficacy* (RADAR-1), constituting the prerequisite that the AI can consistently process and analyze CT brain images for subsequent tasks. *Diagnostic accuracy efficacy* (RADAR-2) is perhaps the most widely reported evidence type in AI literature. In our stroke example, this could pertain to the sensitivity and specificity of the algorithm in finding and highlighting large vessel occlusions. Both RADAR-1 and RADAR-2 are foundational measures, addressed before clinical implementation. Adequate diagnostic accuracy (RADAR-2) could allow for an impact on *diagnostic thinking* (RADAR-3) if the radiologist’s diagnostic workflow changes due to AI usage (for instance, when utilizing AI speeds up the stroke-diagnosis workflow). An impact on the *therapeutic process* (RADAR-4) occurs when, e.g., accurate and fast stroke diagnosis results in more thrombectomies performed. Efficacy in the first four levels culminates in actual *patient outcomes* (RADAR-5), which could in our example be measured as a reduction in long-term brain damage.

Whereas RADAR-1–5 are mostly clinically oriented, *cost-effectiveness efficacy* (RADAR-6) expands to incorporate wider considerations by contrasting costs with societal health benefits. Finally, the added level of *local efficacy* (RADAR-7) highlights the local adaptability and feasibility of the technology, for instance, the fit to the workflow of a specific hospital or stroke center.

## RADAR-1 through RADAR-5: the assessment of clinical value

The first five RADAR levels predominantly pertain to clinical value, starting from technical efficacy (RADAR-1) and culminating in broad patient outcome efficacy (RADAR-5). The appropriate clinical valuation method should conform to the AI system’s objective, typically aligning with one of three primary aims: description, identification, or explanation [15].

Descriptive studies shed light on disease frequency without causal considerations [16], mostly irrelevant in radiology AI. Identification studies discern individuals with a disease (diagnostic) or those at risk (prognostic) [17], the first of which we focus on as it mostly pertains to radiology AI. In this light, we discuss the *cross-sectional study* and in silico* clinical trial (IST)* focused on RADAR-1 and RADAR-2. Finally, we also focus on explanation-based studies exploring causality and the mechanisms of the AI system’s impact. Against this background, we delineate the *randomized controlled trial (RCT)* and observational *cohort study*, related to RADAR-3 through RADAR-5. All discussed study designs are summarized in Table 2.

**Table 2:** Overview of the study designs for the assessment of clinical value in radiology AI (RADAR-1 through RADAR-5)

Attribute	Cross-sectional study	In silico clinical trial	Randomized controlled trial	Cohort study
General description	Study design that analyzes data collected from a population, or a representative subset	Simulations of clinical trials using patient data	Study design where participants are randomly assigned to groups, typically an AI strategy and control group	Study design in which AI-exposed and non-exposed are followed over time for specific outcomes
Type of method/study design	Observational	Experimental	Experimental	Observational
Type of research question	Identification of disease (diagnostic)	Identification of disease (diagnostic)	Explanation (causation) of impact AI as opposed to standard of care	Explanation (causation) of impact AI as opposed to the standard of care or identification of disease (diagnostic)
Time frame	Instantaneous	Instantaneous to longitudinal (simulated over time)	Longitudinal	Longitudinal
Primary outcome	Efficacy of AI in diagnosing conditions (e.g., sensitivity, specificity)	Efficacy of AI in diagnosing conditions (e.g., sensitivity, specificity)	Differences in patient outcomes between treatment and control groups	Differences in patient outcomes between AI and non-AI groups
Example	Cross-sectional study of AI for predicting readmission or death after ICU discharge [45]	IST of digital breast tomosynthesis as a replacement for full-field digital mammography [46]	RCT of decision support algorithm for neonatal seizure recognition [47]	Cohort study of AI solution for referable thoracic abnormalities on chest radiography [48]

*Abbreviations*: *AI* Artificial intelligence, *ICU* Intensive care unit, *RCT* Randomized controlled trial, *SoC* Standard of care

### Cross-sectional study

In the valuation of radiology AI, cross-sectional studies—single-point-in-time studies that assess a specific variable or outcome without requiring long-term follow-up—serve as a useful design [16]. They could assess whether the AI system is technically efficacious (RADAR-1), e.g., in assessing the technology’s capabilities in accurately interpreting radiographic images. Cross-sectional studies could also measure the technology’s efficacy in diagnosing patients with a certain condition (RADAR-2), for instance, in terms of sensitivity and specificity in identifying lung nodules from CT scans. Cross-sectional studies are relatively fast and cheap, as they require only a single interaction with the study population and no time-consuming follow-up.

Their design does not afford a longitudinal perspective, limiting insights into the radiology AI’s performance over extended periods. Therefore, cross-sectional studies are less equipped for addressing the AI’s influence on treatment decisions or patient outcomes (RADAR-3 through RADAR-5), as these often necessitate a longitudinal design (e.g., RCT or cohort study).

### In silico clinical trial

For radiology AI, there is commonly a big gap between retrospective proof-of-concept studies (RADAR-1) and a solution robustly evaluated in a clinical setting (RADAR-3 to RADAR-5). Retrospective studies in radiology AI generally focus on technical efficacy, while other aspects are equally crucial for trustworthy AI (e.g., fairness, usability, robustness) [18]. While RCTs are considered the golden standard to overcome this gap, conducting an RCT for every radiology AI is time-intensive and costly.

To this end, virtual or in silico clinical trials (ISTs) have been proposed. ISTs assess the initial viability and potential of a technology, functioning as a preparatory step for RCTs [19–21]. The main difference is that, instead of human subjects, digital data is used. ISTs are therefore easier to organize, less expensive, and have a lower entry level compared to RCTs. To ensure high levels of evidence before transitioning into RCTs, guidelines for ISTs are continuously evolving and becoming stricter to mimic RCTs as closely as possible.

In addressing technical efficacy (RADAR-1), ISTs might for instance be used to simulate the extent to which an AI technology can process X-ray scans in bone fractures. Moreover, they can offer insight into diagnostic accuracy (RADAR-2), for instance, through modeling the technology’s proficiency in finding long nodules in chest CTs. Furthermore, since ISTs can emulate various clinical situations, they could for example mimic the AI recommendation’s influence on the radiologist’s assessment of detecting irregularities in initial breast mammograms, addressing its influence on clinical management decisions (RADAR-3) before more comprehensive examination in a later-stage study. Pending further advancements, prospective ISTs could theoretically also address RADAR-4 and RADAR-5.

The idea of ISTs for healthcare, already proposed in 2011, has only recently been applied to radiology AI, largely due to the challenges in digital data generation [22]. As the assumption of ISTs is that the results of digital data generalize to real patient data, the generation of representative and realistic digital data is crucial for the validity of ISTs. Two prevailing approaches are virtual patient generation from compiled datasets and the use of digital twins mimicking individual patients [23, 24]. Technological advancements have eased data simulation and improved generalization to real patient data. Yet, each method requires specific developments and stringent quality control for accurate representation.

### Randomized controlled trial

RCTs are underrepresented in (radiology) AI [25, 26], which aligns with the absence of careful value assessment [26]. RCTs are widely recognized as the gold standard in evidence-based medicine and could strongly benefit radiology AI valuation. In terms of diagnostic thinking efficacy (RADAR-3), RCTs investigate if there is a shift in the radiologist’s diagnostic process and whether such changes yield measurable improvements. Regarding therapeutic efficacy (RADAR-4), RCTs measure the effect of AI on treatment strategies, such as how AI assistance in interpreting images affects the final choice of treatment. In terms of patient outcomes (RADAR-5), they might finally evaluate direct patient outcomes, such as whether the AI-guided intervention results in improved survival rates.

To draw adequate causal conclusions, researchers must maximize internal validity. RCTs are highly regarded due to their robust internal validity, which is maximized when selection bias, information bias, and confounding bias are mitigated. Selection bias (i.e., the relation between inclusion in the study and exposure assignment) is minimized through exposure assignment after individuals are included in the study, information bias through (double) blinding, and confounding bias through randomization, which ensures a balanced distribution of potential confounders across the exposed and unexposed arms. While RCTs boast strong internal validity, their external validity (or generalizability) can be a concern due to strict eligibility criteria, which limit applicability to certain populations outside the controlled setting. Improving external validity in RCTs is challenging and would generally rely on replicating the study with a wider scope of patients (e.g., through a multicenter approach).

### Cohort studies

In a systematic review on AI in clinical radiology, 98% of clinical questions were approached with (retrospective) cohort studies, making them easily the most employed study design [27]. Cohort studies investigate associations between intervention and outcomes over time, with participants compared by exposure status. Although fundamentally longitudinal, a single measurement instance can also facilitate a cross-sectional study, allowing for both explanatory and identification-based research questions to be addressed.

While RCTs are often considered the gold standard, cohort studies provide a viable alternative. Opposed to the high costs and limited duration of RCTs, cohort studies can follow larger populations over extended periods, focusing on the long-term effects of AI on patient health outcomes (RADAR-5). Cohort studies often allow for a large study population, resulting in strong external validity.

In conducting a cohort study, one must however address potential threats to internal validity, including selection, information, and confounding biases. Yet, with careful design and analysis, these issues can be anticipated. Emergent analytic techniques, such as instrumental variables (like Mendelian randomization in genetics), generalized methods (e.g., *g*-formula, structural models), and target-trial emulations offer accurate causal inferences. For instance, target-trial emulation can simulate an RCT within a cohort study, offering insight into AI impacts without the necessity to repeatedly conduct expensive and time-consuming RCTs.

## RADAR-6 and RADAR-7: the assessment beyond clinical value

### Health economic evaluations

Health economic evaluations (HEEs) are vital in understanding the (societal) financial feasibility of health technologies, yet are notably scarce in medical AI [28, 29]. HEEs contrast costs and health outcomes of two or more technologies, such as comparing an AI technology with the standard of care [30]. Costs encompass direct expenditures like purchasing, licensing, and training costs, as well as indirect costs such as productivity loss and informal care costs. Outcomes are typically patient (health) outcomes such as quality-adjusted life-years (QALYs) (RADAR-5), customarily obtained from RCTs, observational studies, modeling, or a combination thereof. HEEs can be leveraged to address cost-effectiveness efficacy (RADAR-6), moving beyond only clinical effectiveness [30].

Table 3 displays three common HEE methods. Cost-minimization analysis (CMA) is utilized when there is sufficient reason to believe that the AI system does not improve (clinical) outcomes but has the potential to reduce costs due to improving the clinical-diagnosis workflow. Cost-effectiveness analysis (CEA) may be utilized if the AI system has the potential to improve the clinical outcomes of patients, providing insight into the ratio of (improved) clinical outcomes and costs, captured in the incremental cost-effectiveness ratio. Cost-utility analysis (CUA) is finally similar to CEA, except that clinical outcomes are measured in quality-adjusted life years, so as to ensure standardized comparisons of technologies across healthcare fields.

**Table 3:** Overview of the health economic evaluation study designs for the assessment of cost-effectiveness efficacy in radiology AI (RADAR-6)

Attribute	Cost-minimization analysis	Cost-effectiveness analysis	Cost-utility analysis
Description	Compares costs of technologies (known or assumed to be equally effective) to find the cheapest option	Compares costs and outcomes of different technologies to assess if any health benefits justify costs	Compares costs and outcomes of different technologies to assess if any QALY benefits justify costs
Key input data	Costs	Costs, health outcomes (such as life expectancy, reduction in blood pressure)	Costs, health outcome in terms of QALYs
Literature example	CMA of lung nodule management strategy leveraging AI in lung cancer CT screening [49]	CEA of AI-based chest CT analysis for rapid COVID-19 diagnosis and prognosis [50]	CUA of AI support in CT-based lung cancer screening [51]

*Abbreviations*: *AI* Artificial intelligence, *BIA* Budget impact analysis, *CEA* Cost-effectiveness analysis, *CMA* Cost-minimization analysis, *CUA* Cost-utility analysis, *QALY* Quality-adjusted life year

### Budget impact analysis

Efficacy determined in the initial RADAR levels may not generalize to every local context, necessitating an evaluation of how well the value identified in RADAR-1 through RADAR-6 translates. For instance, local variation in workflow, population composition, and IT infrastructure can all affect the ultimate value of an AI technology and thereby acquisition [31]. It is thus vital to customize AI valuation to align with the specific features and requirements of the local healthcare settings captured in RADAR-7.

To address local financial feasibility, budget impact analysis (BIA) evaluates the AI by considering local budgetary constraints and local population composition (Table 4) [32]. A comprehensive BIA accounts for locally estimated costs, including acquisition, maintenance, training, and workflow adaptation. This provides valuable insights into the AI’s affordability and sustainability for local radiological practices and aids in optimal decision-making during the acquisition phase.

**Table 4:** Overview of the study designs for the assessment of local efficacy in radiology AI (RADAR-7)

Attribute	Budget impact analysis	Multi-criteria decision analysis
Description	Estimates the financial consequences of adopting a technology in terms of affordability and budgetary planning	Evaluates alternatives by considering multiple criteria simultaneously, wherein decision-makers allocate weights to each criterion
Time frame	Short to medium-term	Flexible, depending on the criteria
Key input data	Costs, savings, budget constraints, uptake	Costs, health outcomes, patient preferences, ethical considerations, etc
Literature example	BIA of radioactive seed localization program for non-palpable breast lesions at Canadian hospital [52]	MCDA of different MRI systems for regional hospitals in the Czech Republic [53]

*Abbreviations*: *BIA* Budget impact analysis, *MCDA* Multi-criteria decision analysis, *MRI* Magnetic response imaging

When performing a BIA, it is crucial to consider not only the financial implications of implementing a new technology, but also who shoulders the costs and who reaps the benefits. While an AI tool may boost one department’s efficiency, its funding could come from another department. For instance, an AI tool used for early stroke detection may be funded by the radiology department. While the radiology department incurs the costs of purchasing and implementing this AI tool, it could be the neurology department that mostly benefits from the improved diagnostic capabilities due to an increase in efficiency and better patient outcomes. This could occur without any increase in their department’s expenditures, which could result in disagreements over funding responsibility between departments. Understanding these budget dynamics is therefore essential when assessing AI value and increasing adoption, as BIA concerns not only the total cost, but also how these are distributed.

### Multi-criteria decision analysis

Whereas the previously discussed methods mostly focus on clinical outcomes and cost-effectiveness, a broader approach to the valuation of local efficacy (RADAR-7) allows for including not only medical and economic considerations, but also legal, social, and ethical ones, the last being particularly relevant in radiological AI [11, 14, 33–35]. Examples of broader issues are usability (how easy is it to use the AI technology), regulation (how well does the AI technology conform with local regulatory guidelines), explainability (to what extent is the AI system’s decision understood by the radiologist), etc.

While crucial in valuing radiology AI, these issues are difficult to operationalize and quantify through the previously discussed methods. Multi-criteria decision analysis (MCDA) offers a solution by facilitating a comparison of a highly diverse range of issues [36]. An MCDA of an AI tool would involve local stakeholders (1) to identify the key criteria including patient outcomes, cost-effectiveness, ethics, social concerns; (2) to score the AI technology against these criteria; and (3) to calculate an aggregate score for informed decision-making and acquisition. This allows for a broad health technology assessment perspective and ensures alignment with local requirements and constraints, effectively addressing local efficacy (RADAR-7).

### Prospective monitoring

Prospective monitoring is vital in maintaining long-term relevance and efficacy at the local level (RADAR-7). Earlier work advocated a structured three-phased approach for successful local AI integration [37, 38]. Initially, the AI operates in “shadow mode,” allowing for safety assessments without affecting clinical decisions. This is followed by a small-scale workflow test, gathering valuable feedback from involved clinicians. In the final stage, the AI becomes fully operational, necessitating ongoing monitoring. This continuous oversight helps counter challenges such as “model drift” [39, 40], where variations in new data inputs could compromise AI performance. Given the comprehensive yet time-consuming nature of RADAR, and especially in lengthy study designs like RCTs, model drift could erode the study’s relevance by its conclusion. Conducting meticulous planning and post-implementation prospective monitoring is therefore essential.

## Discussion

The RADAR framework is positioned to progressively value radiology AI through seven hierarchical efficacy levels and has been adapted from Fryback and Thornbury’s (1991) imaging efficacy framework [10]. We have expanded this original framework by tailoring it to radiology AI, adding a local efficacy level and connecting the levels with various study methodologies. We thereby provide radiologists and researchers with a framework that helps to conceptualize the valuation of radiology AI throughout the entire lifecycle. Local decision-makers can moreover use RADAR in making well-founded, evidence-based decisions in the acquisition of radiology AI.

While we predominantly showcased RADAR through examples focused on improving patient health outcomes, it is important to note that many AI systems target non-clinical tasks, e.g., the automation of routine administrative work with large language model technologies. RADAR is also positioned to address such AI systems. While in these examples cost savings (RADAR-6) are likely to be most relevant, influence on other RADAR levels is not exempt. For instance, the reduced administrative load could indirectly influence diagnostic thinking (RADAR-3) by granting radiologists more time for precise diagnoses, which could progressively influence the higher RADAR levels. Radiologists and decision-makers should therefore hierarchically progress through all RADAR levels when ascertaining value. Nevertheless, this process is likely to be faster for technologies focused on administrative tasks such as the aforementioned.

Several frameworks have previously been suggested for valuing radiology AI. The international FUTURE-AI consortium has formulated broad principles with an accompanying checklist to guide developers towards creating safe and trustworthy radiology AI [18]. The Canadian Association of Radiologists [41] and Park et al. [42] proposed guidance on addressing technical performance (RADAR-2). Omoumi et al. offered a more comprehensive checklist, assessing the value of radiology AI technologies through a wider array of concerns [43].

RADAR is unique in that it accounts for different valuation needs throughout the radiology AI lifecycle. For instance, early proof-of-concept technologies would mostly require technical efficacy (RADAR-1) and diagnostic (RADAR-2) efficacy to confirm their foundational capabilities. In contrast, further developed technologies, for which cost-effectiveness has been proven (RADAR-6), require local value assessment or a prospective monitoring plan (RADAR-7) to ensure their broader utility translates locally. RADAR is thus contingent on the state and valuation need of the specific technology, which is vital as this changes throughout the radiology AI lifecycle.

In conclusion, RADAR constitutes a conceptual framework for the valuation of radiology AI throughout its lifecycle. It initiates with technical performance at the technology’s conception (RADAR-1) and incorporates increasingly broader valuation, ultimately resulting in the assessment of generalizability to the local context (RADAR-7). Progressing hierarchically through the seven levels, RADAR constitutes a comprehensive valuation framework, positioned to bridge the implementation gap in radiology AI.

## Data Availability

Not applicable.

## Abbreviations

AI: Artificial intelligence
BIA: Budget impact analysis
CEA: Cost-effectiveness analysis
CMA: Cost-minimization analysis
CUA: Cost-utility analysis
ESR: European Society of Radiology
HEE: Health economic evaluation
IST: In silico clinical trial
MCDA: Multi-criteria decision analysis
QALY: Quality-adjusted life year
RADAR: Radiology AI Deployment and Assessment Rubric
RCT: Randomized controlled trial
